# Fluconazole resistance and CDR1 expression in Candida albicans mediated by the hyperactive Tac1-5 transcriptional activator requires Tlo proteins

**DOI:** 10.1099/mic.0.001594

**Published:** 2025-09-01

**Authors:** Wen Jun Lim, Brenda Lee, Zahra Farrington, Abed Alkarem Abu Alhaija, Alastair B. Fleming, Derek J. Sullivan, Gary P. Moran

**Affiliations:** 1 Division of Oral Biosciences, Dublin Dental University Hospital, University of Dublin, Trinity College Dublin, Dublin, Ireland; 2 Department of Microbiology, School of Genetics and Microbiology, University of Dublin, Trinity College Dublin, Dublin, Ireland

**Keywords:** *Candida albicans*, CDR1, fluconazole, resistance, TAC1, TLO

## Abstract

*Candida albicans* is an opportunistic fungal pathogen associated with superficial and systemic infections in humans. Azole antifungal resistance in *C. albicans* is of clinical concern, and both oral and systemic *Candida* infections can be difficult to treat due to the lack of alternative antifungal drugs. Expression of a hyperactive form of the transcription factor Tac1 is a major contributor to azole resistance in *C. albicans* isolates resulting in the increased expression of the azole efflux pump Cdr1. In this study, we investigated whether the Mediator tail component Med2, encoded by the expanded (n=14) *TLO* gene family of * C. albicans*, was required for Tac1 activity. A homozygous *TAC1-5* gain-of-function point mutation was introduced into WT, *tlo*Δ and *med3*Δ strains of *C. albicans* which enables them to express hyperactive Tac1. qRT-PCR analysis revealed that *tlo*Δ-*TAC1-5* had reduced basal and fluphenazine-induced *CDR1* expression relative to WT-*TAC1-5* strains and exhibited reduced levels of resistance to fluconazole and terbinafine. Individual copies of representatives from each of the alpha, beta and gamma *TLO* clades were reintroduced into *tlo*Δ-*TAC1-5* to investigate their ability to restore Tac1-activated resistance. These studies show that alpha and beta *TLO* genes could restore fluconazole resistance in the *tlo*Δ-*TAC1-5* background, whereas gamma clade genes did not result in any detectable phenotypic complementation. Transcript profiling showed that reintroduction of *TLO*α*1* led to increased expression of *TAC1-5*-activated genes such as *CDR1*. Further analysis using ChIP-qPCR revealed that Tloα1 localizes to the drug response element which is the site where Tac1 binds to the *CDR1* promoter. These data have identified that the *TLO* gene family is required for the expression of Tac1-mediated fluconazole resistance. However, this effect is confined to members of the alpha and beta, but not the gamma, *TLO* clades.

## Data Availability

All RNAseq data are available from the NCBI Sequence Read Archive, submission number SUB15226071, under the accession numbers SRX28236589, SRX28236588, SRX28236587, SRX28236586, SRX28236585, SRX28236584, SRX28236583, SRX28236582, SRX28236581, SRX28236580, SRX28236579 and SRX28236578.

## Introduction

*Candida albicans* is an opportunistic pathogenic yeast species and is the major cause of superficial candidiasis and candidemia globally [1,2]. The World Health Organization has recently designated *C. albicans* as a fungal priority pathogen [3,4]. Surveillance reports suggest that resistance to the commonly used azole antifungal drugs, including fluconazole, continues to be a concern when treating *C. albicans* infections [5,7]. Multiple factors have been identified that may contribute to the increased incidence of azole-resistant *C. albicans*, such as the wide and long-term usage and the fungistatic nature of azole drugs [1,2].

The most common mechanisms of azole resistance in *C. albicans* are the alteration of the drug target enzyme (Erg11), the overexpression of Erg11 and the upregulation of plasma membrane efflux pumps [8]. Most azole-resistant isolates of *C. albicans* exhibit increased expression of multidrug transporters encoded by either the major facilitator efflux pump *CaMDR1* (multidrug resistance 1) or the ATP-binding cassette (ABC) transporters *CDR1* (*Candida* drug resistance) and *CDR2* [9]. Furthermore, gain-of-function (GOF) mutations in the transcriptional activator Tac1 are commonly responsible for the overexpression of efflux pumps in these isolates [9,10]. Tac1 is a classical zinc cluster transcription factor that possesses a highly conserved Zn_2_Cys_6_ motif [11]. Tac1 has three domains, an N-terminal DNA-binding domain (DBD; 1–1,320 nucleotides), a C-terminal acidic activation domain (AAD; 1,354–2,946 nucleotides) and a middle homology region (MHR; 1,321–1,653 nucleotides) [11,12]. The Tac1-DBD binds to the drug response element (DRE) in the promoter regions of *CDR1* and *CDR2* [11]. Tac1-MHR is known to be a xenobiotic-binding domain that interacts with xenobiotics including fluphenazine, resulting in Tac1 hyperactivity and upregulation of *CDR1* and *CDR2* expression [11]. The Tac1-AAD was shown to interact with the TATA-binding protein (TBP) that plays a crucial role in transcription [11]. The well-studied *TAC1*^GOF^ mutation identified in a *C. albicans* azole-resistant isolate, known as *TAC1-5*, is a nonsynonymous substitution of Asn to Asp in Tac1p [9]. It is located within the Tac1-AAD, and it has been suggested that this substitution enhances the interaction between Tac1-AAD and TBP, thus increasing the transcription of *CDR1* and *CDR2* [12].

The Mediator multi-polypeptide complex is an important component in transcription initiation and has been shown to regulate the transcription of various antifungal resistance-related mechanisms. In *C. albicans*, Mediator tail subunits Med3 and Med15 have both been shown to be required for Tac1-activated azole resistance and xenobiotic induction of *CDR1* expression [13]. In *Candida glabrata*, the Mediator tail components CgMed15A and CgMed2 are required for the regulation of expression of the ABC transporter-encoding genes Cg*CDR1* and Cg*CDR2* (orthologues of *C. albicans CDR1/2*) via interaction with the transcription factor CgPdr1 [14,15]. The Mediator complex in *C. albicans* differs from most other species due to the large expansion of genes encoding the Med2 tail component, encoded by a 10–15 member gene family, referred to as the *TLO* family [16,17]. Phylogenetic analysis divides the gene family into three distinct clades, and previous studies suggest that the *TLO* genes/clades play different roles in controlling the expression of a wide range of cellular functions, including virulence and tolerance to azole drugs [18,22]. Given the role of the Mediator tail components Med3 and Med15 in Tac1-activated *CDR1* expression and azole resistance in *C. albicans*, we wanted to investigate if members of the expanded *TLO* gene family (which encode the Med2 Mediator tail component) are required for basal *CDR1* expression and Tac1-activated *CDR1* expression in *C. albicans* by introducing the homozygous *TAC1-5* allele (N977D substitution) into a *tlo*Δ mutant (in which the entire *TLO* family had been deleted previously).

## Methods

### *Candida* strains and growth conditions

All *C. albicans* strains used in this study, including CRISPR-Cas9-edited strains, are described in Table S1 (available in the online Supplementary Material). All *C. albicans* strains were routinely grown on yeast extract peptone dextrose (YEPD) agar at 30 or 37 °C, as indicated, for 24–48 h in a static incubator (Gallenkamp, Leicester, UK). For liquid culture, *C. albicans* was routinely grown in YEPD broth in a shaking incubator (New Brunswick Scientific, Edison, NJ, USA) set at 200 r.p.m., 30 or 37 °C overnight. Nourseothricin-resistant strains were selected and maintained on YEPD medium containing 200 µg ml^−1^ of the antibiotic (CloNAT, Werner Bioagents, Germany).

### Antifungal susceptibility testing

Fluconazole spot plate assays were performed on YEPD plates with or without fluconazole (10 or 15 µg ml^−1^). Plates were inoculated with 5 µl spots of serial dilutions of overnight cultures (2×10^6^ to 2×10^2^ cells ml^−1^). Plates were incubated in a static incubator for 48 h at 30 ˚C. Fluconazole disc diffusion assays and Etests were performed on solid RPMI-1640 medium, prepared from liquid RPMI-1640 medium (Merck) as described by O’Connor-Moneley *et al.* [21]. Overnight YEPD broth cultures were washed with sterile PBS and resuspended to OD_600_=0.07. Sterile cotton swabs were dipped in the suspension and used to lawn solid RPMI-1640 agar plates. A 25 µg fluconazole susceptibility disc (OXOID), or fluconazole Etest (bioMérieux), was placed at the centre of each RPMI-1640 agar plate and incubated at 30 ˚C for 48 h. The plates were imaged on a ‘FLASH and GO’ plate imager (IUL Instruments) using autoexposure settings.

### Design and construction of the CRISPR-Cas9 oligonucleotides

The CRISPR-Cas9 LEUpOUT system designed for *C. albicans* [23] was used to introduce a homozygous *TAC1-5* SNP (an A to G substitution at nucleotide position 2929) into the *C. albicans* strain MAY1244 (*LEU2/leu2*Δ) and its *tlo*Δ and *med3*Δ derivatives. A unique 20 bp gRNA (Table S2) was designed to cut *TAC1* within the TAD, and a repair template (94 bp length, generated from overlapping oligonucleotides; Table S2) containing the A to G substitution at nucleotide position 2929 was introduced. The gRNA-expressing cassette was generated by a fusion PCR using oligonucleotides and plasmids previously described [23]. Transformation into *C. albicans* was performed using the electroporation method [24]. *C. albicans* transformants with homozygous *TAC1-5* mutations were identified via Sanger sequencing following amplification of the region with the primers TAC1ampF and TAC1-5 R (Table S2) yielding a 440 bp DNA product. The recycling of the Cas9 cassette was carried out on the same day as PCR streaking colonies on YNB plates without amino acids or ammonium sulphate (leucine-negative media) and incubation at 30 °C for 2–3 days. Colonies that grew on plates without leucine and exhibited nourseothricin sensitivity were considered to have excised the CRISPR-Cas9 components from the *LEU2* locus.

### RNA extraction and qRT-PCR analysis

YEPD broth cultures were inoculated from overnight cultures to an OD_600_=0.1 and incubated at 37 °C with 200 r.p.m. shaking until an OD_600_=0.8 was reached. RNA was then extracted from cells using the RNeasy extraction kit (Qiagen) as per the manufacturer’s instructions. mRNA sequencing was performed with strand-specific libraries and sequenced on the Illumina NovaSeq 6000 Sequencing System using paired-end 150 bp reads. Each experiment generated a minimum of >20 million read pairs per sample with a Q30 score of ≥85%. Raw reads were aligned to the *C. albicans* SC5314 Assembly 21 genome (downloaded from CGD) in the Strand NGS 4.0 software package using the default settings. Reads were quantified and normalized in Strand NGS using DeSeq2 [25], and statistical analysis of differential expression was carried out with post hoc Benjamini–Hochberg testing (FDR q<0.05). Further analysis on lists of differentially expressed genes was performed via GO analysis on the *Candida* Genome Database and Gene Set Enrichment Analysis (GSEA) [26]. For GSEA, a custom database of *C. albicans* genes associated with GO Terms and previously published RNA sequencing experiments was queried (Supplementary data file). Sequence data are available for download from the NCBI Sequence Read Archive, accession PRJNA1245622.

For qRT-PCR analysis, *Candida* cultures were established as described above in 100 ml YEPD (OD_600_ 0.1) and grown to exponential phase (OD_600_ 0.6–0.8) in a shaking incubator set at 200 r.p.m., 37 °C. An 80 ml volume of mid-log phase *C. albicans* culture was then split equally into two conical flasks, with one being exposed to fluphenazine at a final concentration of 20 µg ml^−1^ and the other as a control (no drugs added). A 10 ml volume of each culture was then collected at given time points (0 and 30 min) and was used for RNA extraction as above. The Superscript IV Reverse Transcriptase kit (Invitrogen) was used to convert RNA into cDNA. For qRT-PCR, reactions consisted of 7.5 µl SYBR Green, 0.375 µl of each forward and reverse primer (10 mM) and 5.75 µl of molecular-grade water. A 14 µl volume of this mixture was combined with 1 µl cDNA (diluted 1 : 10 in water) as the template. All qRT-PCR primer sequences are listed in Table S2. qRT-PCR reactions were performed in an AB7500 Real Time PCR machine (Applied Biosystems) with each biological replicate split into three technical replicates. The *ACT1* housekeeping gene was used as an endogenous control to determine relative expression using the comparative C_T_ method and graphically represented using GraphPrism v10 (San Diego, CA, USA).

### Reintroduction of *TLO* genes using pNIM1

Individual HA-tagged alpha, beta and gamma *TLO* genes were reintroduced into the *ADH1* locus of the *tlo*Δ-*TAC1-5* mutants using the pNIM1-*TLO* cassettes developed by Fletcher *et al.* [19]. The plasmid was linearized for transformation purposes using *Kpn*I and *Sac*II restriction enzymes (New England Biolabs). Integration of the pNIM1 cassette containing the specific *TLO* gene was confirmed using the *ADH1* locus-specific primer AAK29 and the pNIM1-specific primer AAK31. The presence of the reintroduced *TLO* gene was confirmed with the *TLO* primer ‘Pan-Tlo’ and the HA-tag reverse primer, which yielded products of different size for each *TLO* gene visualized by gel electrophoresis (Table S2). The expression of each reintroduced *TLO* gene in the *tlo*Δ-*TAC1-5* background was measured by qRT-PCR using gene-specific primers listed in Table S2. Further verification was performed using Western blotting against HA-protein to visualize HA-tagged Tlo proteins.

### ChIP-qPCR

*C. albicans* cultures were grown with shaking at 200 r.p.m. and 37 °C in 50 ml YEPD. After reaching OD_600_ 1.0, the cells were washed in PBS and resuspended in 36 ml PBS and 1 ml of 37% (v/v) formaldehyde. Cultures were incubated at 30 °C with shaking at 200 r.p.m. for 30 min, with subsequent quenching with 2 ml sterile 2.5 M glycine for 10 min. Cells were then pelleted and flash-frozen in liquid nitrogen, followed by storage at −80 °C for future use. The pellet containing formaldehyde-fixed cells was defrosted on ice, and 400 µl formaldehyde-assisted (FA) lysis buffer (50 mM HEPES pH 7.5, 140 mM NaCl, 1 mM EDTA, 1% Triton X-100, 0.1% sodium deoxycholate) was added followed by 4 µl PI (1 : 100), 3.2 µl PMSF (2 mM) and an equal volume of zirconia beads (0.5 mm in size, ~500 µl). The resuspended mixture was processed for seven cycles (30 s each) using a FastPrep Homogeniser 24 (4 m/s) and chilled for 1 min on ice between each cycle. The lysate was mixed with 1 ml FA lysis buffer and spun at low speed (8,000 r.p.m.) for 30 s to avoid pellet formation. An 80 µl ‘pre-sonication’ sample was removed after this centrifugation. The remaining centrifuged lysate was gently resuspended and sonicated 14 times (10 s pulses at 8 microns, Sanyo Soniprep 150) with 1 min on ice between each sonication. After sonication, samples were centrifuged at 13,200 r.p.m. for 30 min at 4 °C. An 80 µl aliquot of supernatant was processed for DNA fragment size verification, and the remaining supernatant was then divided into 100 µl aliquots and stored at −80 °C for future use. DNA fragment size was verified by agarose gel electrophoresis.

Prior to analysis, the samples of sonicated lysate were thawed and brought to a volume of 500 µl with FA lysis buffer containing protease inhibitors (PIs) (15 ml FA lysis buffer+150 µl 100X PI, made fresh). The solution was mixed, and 20 µl of lysate was transferred to a PCR tube as the input (IN) sample (Input). Pronase treatment of IN sample was then carried out with the addition of 100 µl ChIP elution buffer, 60 µl TE (pH7.5), 20 µl Pronase (20 mg ml^−1^) and 1 µl 1 M CaCl_2_ with incubation in a thermocycler (42 °C for 2 h, 65 °C for 6 h) before storage at −20 °C. To the remaining 480 µl lysate, 2 µl anti-HA antibody (Abcam ab9110) was added and placed on a rotating wheel at 4 °C overnight at 16 r.p.m. Antibody–DNA complexes were purified using a mix containing 20 µl Protein A and 20 µl Protein G beads (Invitrogen Dynabeads) according to the manufacturer’s instructions. Purified bead supernatants (250 µl) from the IP pulldown samples were subjected to treatment with 50 µl Pronase (20 mg ml^−1^) and 1.5 µl 1M CaCl_2_ with incubation in a thermocycler (42 °C for 2 h, 65 °C for 6 h).

All IN samples and IP pulldown samples were purified with the Qiagen PCR purification kit. qPCR was carried out using the GoTaq^®^ qPCR kit from Promega using IP pulldown samples (diluted 1 in 5) and IN samples (diluted 1 in 50) as DNA template. A serial tenfold dilution (neat to 10^−4^) of DNA (200 ng µl^−1^) isolated from *C. albicans* MAY1244-*TAC1-5* was used to generate a DNA standard curve. A 0.12 µl volume of primer mix containing forward and reverse primer (50 µM), 2× SYBR master mix and 9.88 µl water was mixed with 2 µl diluted DNA for each GoTaq qRT-PCR reaction. Reactions for each biological replicate experiment were run in technical triplicate, for 40 cycles of 95 °C for 15 s and 60 °C for 1 min. Target enrichment in the IP sample (IP/IN) was normalized to the IP/IN signal detected at an internal negative control site (*CDR1* Up, a region ∼1.4 kb upstream of the *CDR1* ORF) and expressed as ‘Relative occupancy’.

## Results

### Introduction of *TAC1-5* hyperactive mutation in *C. albicans* Mediator tail mutants

CRISPR-Cas9-mediated mutagenesis was used to introduce a homozygous *TAC1-5* point mutation in a *tlo*Δ mutant in which the entire *TLO* gene family (*n*=14) had previously been deleted [19], and for comparative purposes, in a *med3*Δ mutant in which the (single-copy) gene encoding the Med3 mediator tail component had been deleted. An identical mutation was also introduced in the parental WT *C. albicans* strain MAY1244 to generate strain WT-*TAC1-5*. Confirmation of the single base pair substitution from A to G at position 2929 bp in both alleles of the *TAC1* gene was confirmed by Sanger sequencing. At the cellular level, the *TAC1-5* derivatives of *tlo*Δ (*tlo*Δ-*TAC1-5*) and *med3*Δ (*med3*Δ-*TAC1-5*) were indistinguishable from the parental mutants, exhibiting a pseudohyphal morphology in YEPD medium and exhibiting an inability to form true hyphae in 10% serum. The WT*-TAC1-5* derivative was morphologically identical to its parental WT isolate (i.e. it grows as yeast in YEPD and forms hyphae in 10% serum).

**Fig. 1.**
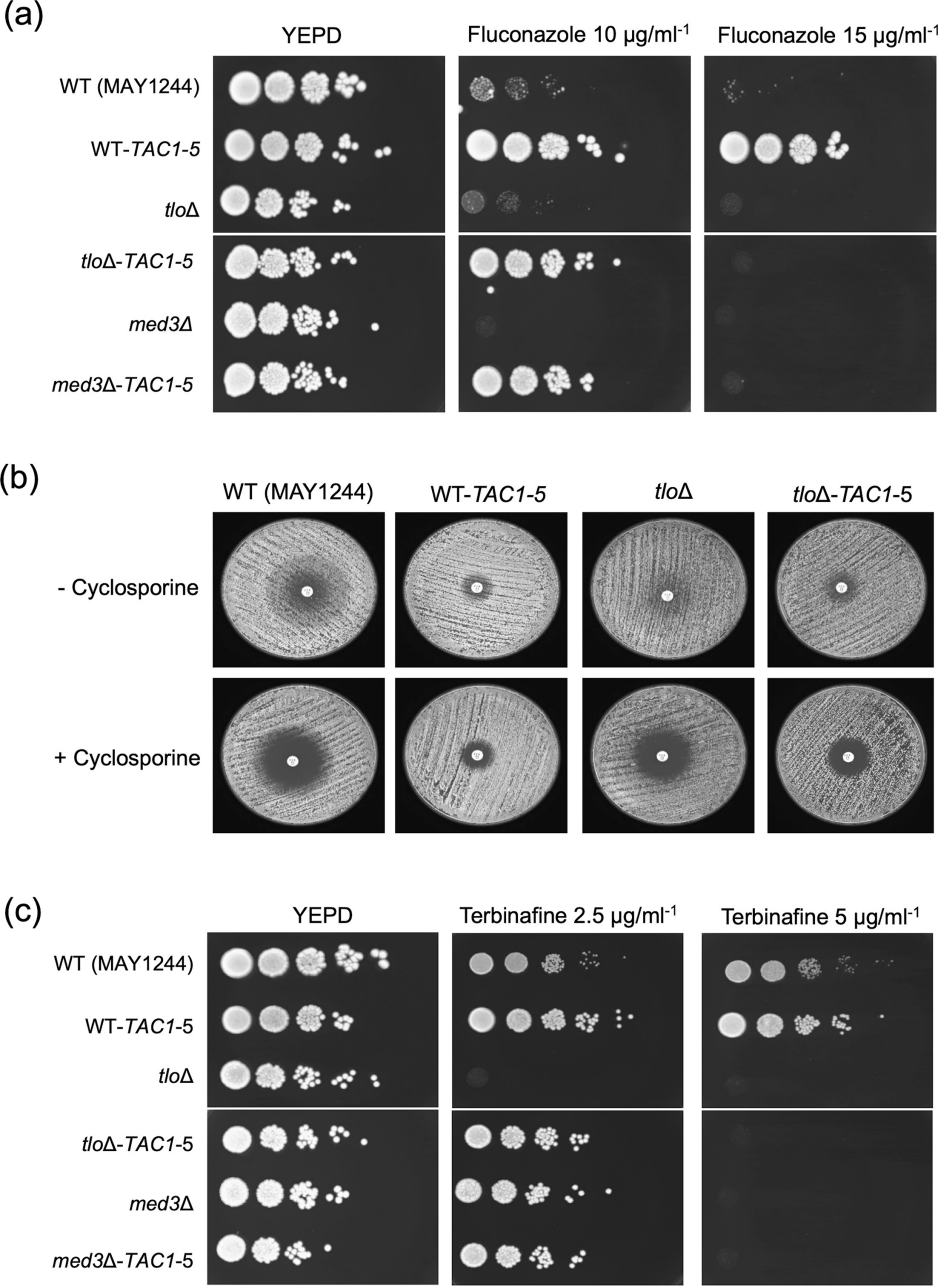
Fluconazole susceptibility of *C. albicans* WT (MAY1244), *tlo*Δ and *med3*Δ null mutants and derivatives expressing the *TAC1-5* GOF mutation. (**a**) Fluconazole spot plate assays were performed by spotting 5 µl of serial dilutions of overnight cultures (2×10^6^ to 2×10^2^ cells ml^−1^) on YEPD plates with or without fluconazole (10 or 15 µg ml^−1^) and incubated for 48 h at 30 °C. (**b**) Disc diffusion assays on RPMI-1640 plates containing fluconazole (25 µg ml^−1^) with and without cyclosporine (1 µg ml^−1^) at 30 °C for 48 h. (**c**) Terbinafine spot plate assays were carried out as in (**a**) on YEPD plates containing terbinafine (2.5 and 5 µg ml^−1^).

As expected, the introduction of the hyperactive Tac1 mutation into the WT parental strain (WT-*TAC1-5*) resulted in resistance to fluconazole (Fig. 1a), with the mutant being able to grow on YEPD spot plates supplemented with 10 µg ml^−1^ and 15 µg ml^−1^ fluconazole. Introduction of the mutation into the *tlo*Δ and *med3*Δ mutants also resulted in reduced susceptibility to fluconazole; however, these strains only grew on plates with 10 µg ml^−1^ but not 15 µg ml^−1^ fluconazole (Fig. 1a). Fluconazole susceptibility was further investigated using a disc diffusion assay (Fig. 1b) in the presence and absence of cyclosporine A (1 µg ml^−1^), to suppress fluconazole tolerance. The introduction of the *TAC1-5* allele conferred increased fluconazole resistance to both WT and *tlo*Δ mutants as they both exhibited smaller zones of inhibition compared to their parental strains (Fig. 1b and Table S3). The WT-*TAC1-5* strain was less susceptible to fluconazole compared to the *tlo*Δ*-TAC1-5* strain, which exhibited a smaller zone of inhibition (3.2 mm reduction, Table S3). As expected from our previous studies, in the absence of cyclosporine, the *tlo*Δ mutant exhibited a high degree of fluconazole tolerance indicated by growth within the zone of inhibition (Fig. 1b). Interestingly, even though the *TAC1-5* mutation conferred increased fluconazole resistance in the *tlo*Δ background, it was found to slightly reduce the fluconazole tolerance of *tlo*Δ, indicated by the clear zone of inhibition (Fig. 1b). Fluconazole Etest assays were also carried out to obtain precise fluconazole MIC values. This analysis showed that the three strains without the *TAC1-5* mutation (MAY1244, *tlo*Δ and *med3*Δ mutants) expressed similar MIC values between 1 and 2 µg ml^−1^ (Table 1). Confirming the spot plate and disc diffusion assays, WT-*TAC1-5* showed the highest fluconazole MIC value (16 µg ml^−1^), while *tlo*Δ**-***TAC1-5* and *med3*Δ**-***TAC1-5* strains exhibited a twofold lower MIC (8 µg ml^−1^).

**Table 1.** Morphological characteristics and fluconazole Etest assay MIC values of *C. albicans* strains and derivatives expressing the *TAC1-5* mutation

Strains		Cellular morphology in YEPD (30 °C)	Morphology in 10% serum	MIC values (Etest)
WT (MAY1244)		Yeast morphology	True hyphae	1.5
WT-*TAC1-5*		Yeast morphology	True hyphae	16
*med3*Δ		Pseudohyphae	Pseudohyphae	1
*med3*Δ*-TAC1-5*		Pseudohyphae	Pseudohyphae	8
*tlo*Δ		Pseudohyphae	Pseudohyphae	1.5
*tlo*Δ*-TAC1-5*		Pseudohyphae	Pseudohyphae	8
*tlo*Δ-*TAC1-5+TLO*α*1*	Alpha clade	Yeast morphology	True hyphae	16
*tlo*Δ-*TAC1-5+TLO*α3	Alpha clade	Yeast morphology	True hyphae	12
*tlo*Δ-*TAC1-5+TLO*α34	Alpha clade	Yeast morphology	True hyphae	16
*tlo*Δ-*TAC1-5+TLO*β*2*	Beta clade	Pseudohyphae	Pseudohyphae	6
*tlo*Δ-*TAC1-5+pENO-TLO*β*2*	Beta clade	Yeasts/hyphae	True hyphae	16
*tlo*Δ *TAC1-5+TLO*γ5	Gamma clade	Pseudohyphae	Pseudohyphae	6
*tlo*Δ *TAC1-5+TLO*γ7	Gamma clade	Pseudohyphae	Pseudohyphae/hyphae	8
*tlo*Δ *TAC1-5+TLO*γ*11*	Gamma clade	Pseudohyphae	Pseudohyphae	8

Sensitivity to the antifungal agent terbinafine, which is also a substrate of the Cdr1 efflux pump, was also assessed in the *C. albicans TAC1-5* mutants on YEPD plates containing 2.5 µg ml^−1^ and 5 µg ml^−1^ terbinafine (Fig. 1c). Both MAY1244 and its derivative WT-*TAC1-5* were capable of growth on 5 µg ml^−1^ terbinafine plates. Surprisingly, although the Δ*med3* mutant was able to grow in the presence of 2.5 µg ml^−1^ terbinafine, the Δ*tlo* mutant was not. However, the introduction of the *TAC1-5* mutation resulted in similar terbinafine resistance profiles as they both exhibited strong growth on the 2.5 µg ml^−1^ terbinafine, but not on 5 µg ml^−1^ terbinafine (Fig. 1c).

### Expression of *CDR1* and *CDR2* in Mediator tail and *TAC1-5* mutants

**Fig. 2.**
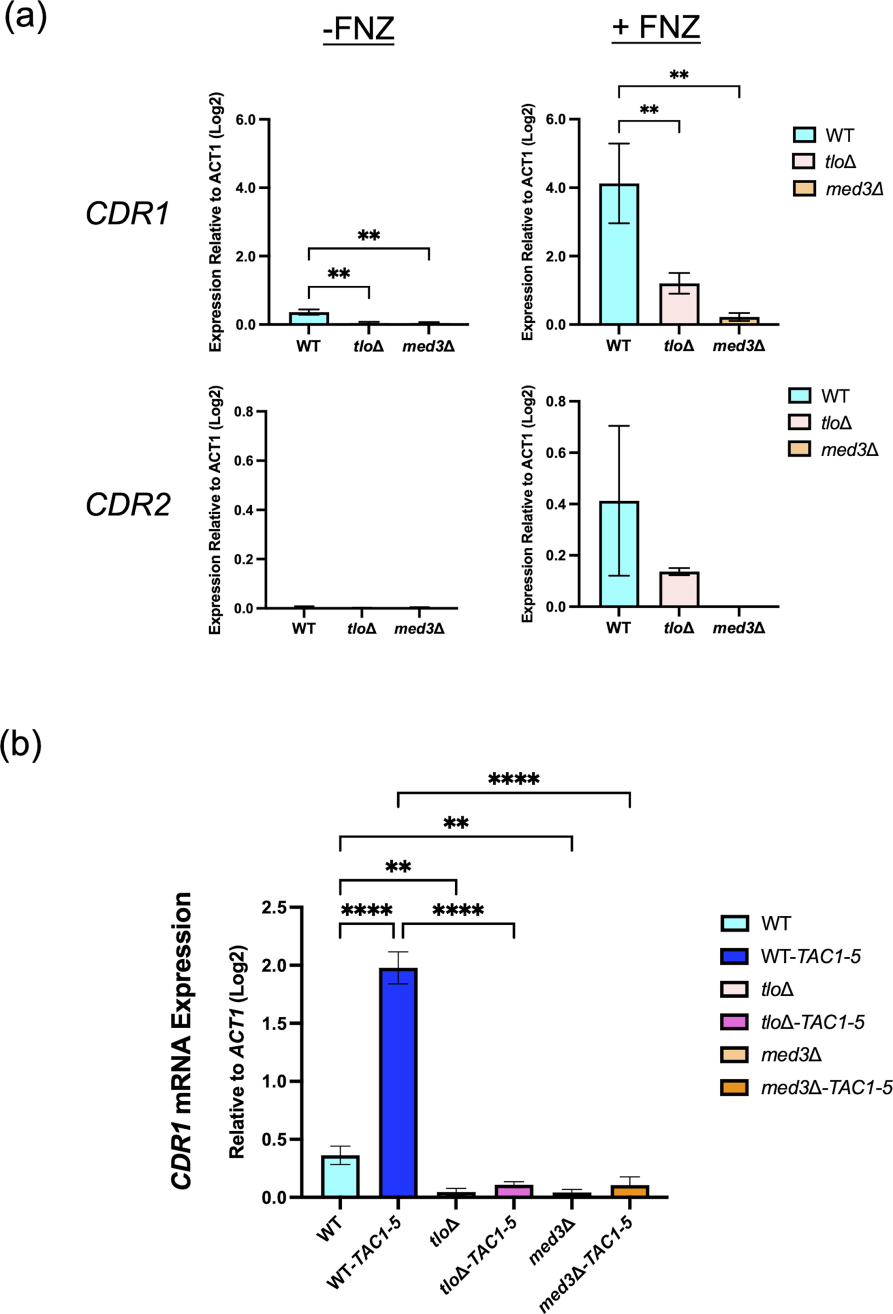
*CDR1* and *CDR2* mRNA expression in *C. albicans* WT and Mediator tail null mutants. (**a**) *CDR1* expression measured via qRT-PCR in YEPD alone (−FNZ) or in the presence of fluphenazine (+FNZ, 20 µg ml^−1^) for 30 min. (**b**) *CDR1* expression in *TAC1-5* derivatives of WT MAY1244, *tlo*Δ and *med3*Δ. The expression of *CDR1* was normalized relative to the expression of *ACT1* and was expressed as a log_2_ fold difference. Error bars were calculated based on three biological replicates, and ordinary one-way ANOVA was carried out to detect any significant differences between strains (* indicates *P* value<0.05, ** indicates *P* value<0.01 and **** indicates *P* value<0.0001).

In order to investigate the expression of *CDR1* and *CDR2,* the WT *C. albicans* and Mediator tail mutant strains were grown in the absence and presence of the xenobiotic fluphenazine for 30 min (Fig. 2a). qRT-PCR revealed that both *tlo*Δ and *med3*Δ mutants exhibit significantly lower basal *CDR1* expression and lower basal *CDR2* expression compared to WT *C. albicans*. Exposure to fluphenazine for 30 min resulted in high-level induction of *CDR1* and *CDR2* mRNA in WT *C. albicans* but only weakly induced the expression of *CDR1* and *CDR2* mRNA in *tlo*Δ and *med3*Δ mutants. As per previous studies, *CDR1* expression was significantly higher than that of *CDR2* in all strains tested [9,10].

As expected, the presence of the homozygous *TAC1-5* mutation significantly increased the expression of *CDR1* in the WT (~4-fold log2; *P*<0.01) strain (Fig. 2b). However, no significant increase in *CDR1* expression was observed in either of the Mediator tail mutants (*tlo*Δ and *med3Δ*; Fig. 2b).

### Reintroduction of *TLO*α*1*, *TLO*β*2* and *TLO*γ*11* in *tlo*Δ-*TAC1-5*

**Fig. 3.**
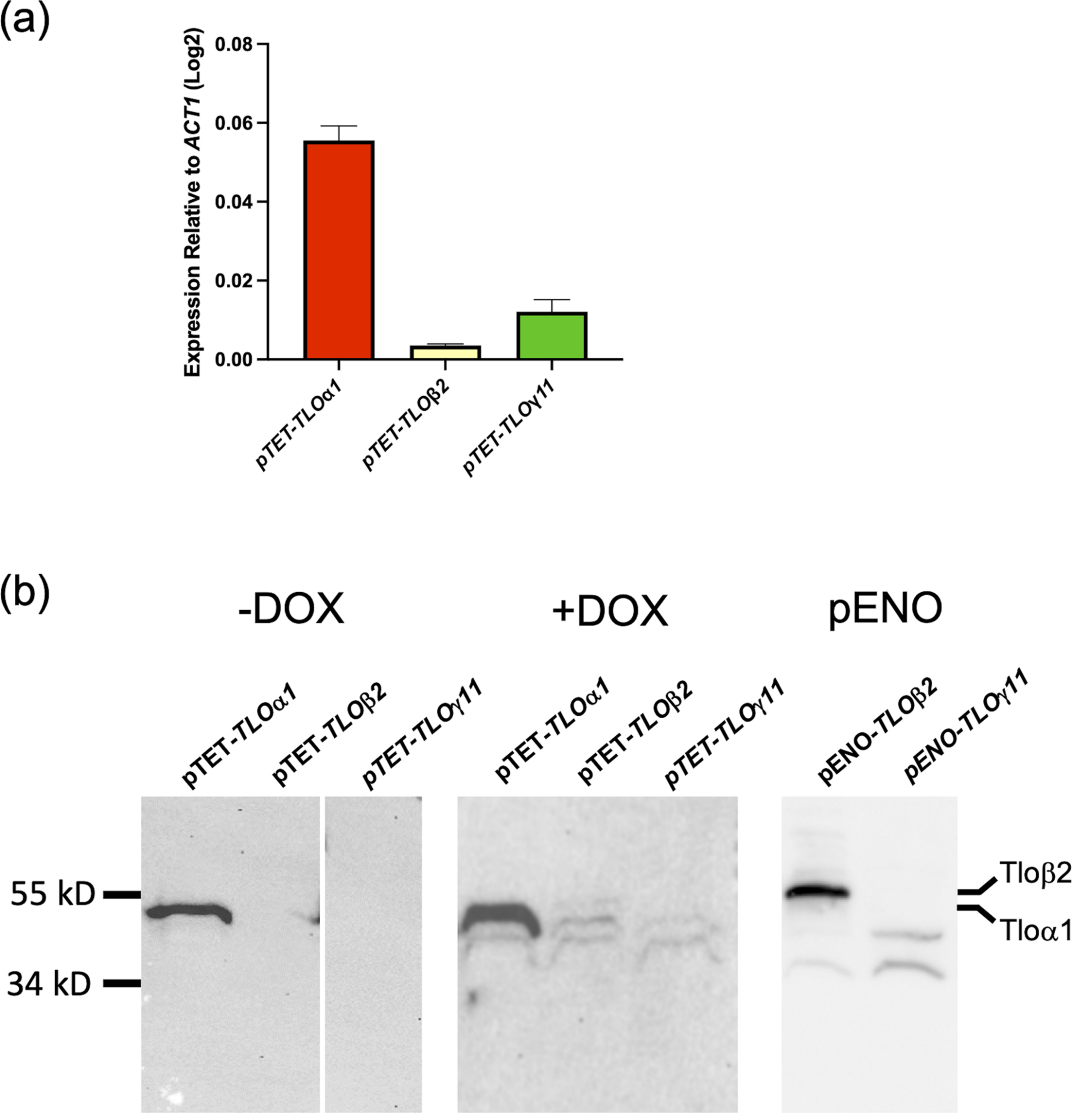
qRT-PCR and Western blot analysis to detect HA-tagged Tlo expression in *tlo*Δ and *tlo*Δ*TAC1-5* mutants expressing individual *TLO*s (*TLO*α*1*, *TLO*β*2* and *TLO*γ*11*). (**a**) *TLO* gene expression was measured by qRT-PCR in mid-exponential YEPD broth cultures. The expression of *TLO* genes was normalized relative to the expression of *ACT1* and was expressed as log_2_ fold difference. (**b**) Crude protein was extracted from exponential phase *tlo*Δ and *tlo*Δ*-TAC1-5* mutants expressing individual *TLO*s (*TLOα1*, *TLO*β*2* or *TLO*γ*11*) grown in YEPD media at 37 ˚C. Anti-HA antibody (ab9110, Abcam) was used to detect HA-tagged Tlo proteins.

In order to investigate if individual *TLO* genes can complement the drug response phenotypes of the Δ*tlo* mutant, we reintroduced representatives from each of the three *TLO* clades (*TLO*α*1*, *TLO*β*2* and *TLO*γ*11*) into the *tlo*Δ-*TAC1-5* mutant under the control of the *pTET* promoter. Expression levels of the *pTET*-regulated *TLO*α*1* mRNA and the Tloα1 protein in the *tlo*Δ-*TAC1-5* strain in doxycycline-free medium were similar to that previously described by Fletcher *et al.* [19] when the same construct was expressed in the parental *tlo*Δ background. Phenotypically, reintroduction of *TLO*α*1* also reversed the pseudohyphal morphology of the *tlo*Δ-*TAC1-5* strain and restored true hypha formation in 10% serum (Fig. S1). Expression of *TLO*β*2* mRNA from the same *pTET* promoter in *tlo*Δ-*TAC1-5* was significantly lower than *TLO*α*1* and also lower than the previously reported levels of expression of *TLO*β*2* when the same construct was expressed in the parental *tlo*Δ background [19] (Fig. 3). Western blotting could not detect expression of HA-tagged Tloβ2 protein in the *tlo*Δ*-TAC1-5* mutant, even following doxycycline induction, and the construct could not complement the cell morphology or hyphal growth defects in the *tlo*Δ-*TAC1-5* background (Fig. S1). Only when *TLO*β*2* was reintroduced under the strong, constitutive enolase promoter (*pENO*) were we able to detect Tloβ2 and observe phenotypic complementation of the cell morphology and hyphal growth defects of the mutant strain, indicating functional Tloβ2 expression (Fig. S1).

Expression levels of *pTET-TLO*γ*11* mRNA in the *tlo*Δ-*TAC1-5* background were similar to that described by Fletcher *et al.* [19]. Despite the presence of detectable levels of mRNA, we could not detect expression of the Tloγ11 protein in the *tlo*Δ-*TAC1-5* background, which concurs with the findings of Fletcher *et al.* [19] who were unable to detect the Tloγ11 protein, even when expressed from the strong *pENO* promoter (Fig. 3b).

### Antifungal susceptibility of *tlo*Δ-*TAC1-5* mutants complemented with *TLO*α*1*, *TLO*β*2* or *TLO*γ*11*

**Fig. 4.**
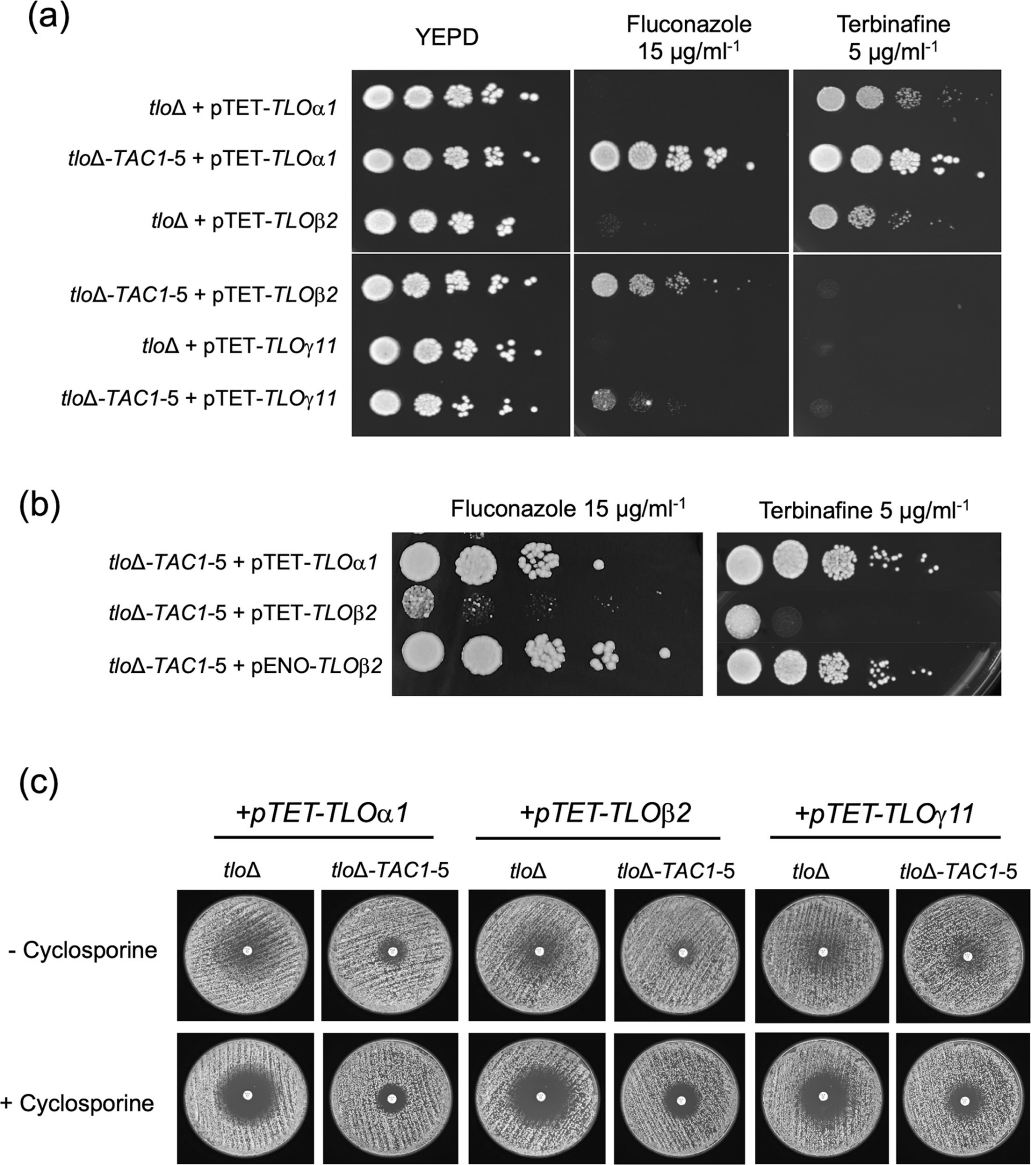
Fluconazole and terbinafine susceptibility of *tlo*Δ and *tlo*Δ*-TAC1-5* mutants complemented with *TLO*α*1*, *TLO*β*2* or *TLO*γ*11*. (**a**) Spot plate assays were inoculated with serial dilutions of overnight cultures (2×10^6^ to 2×10^2^ cells ml^−1^) on YEPD plates with or without fluconazole or terbinafine. Plates were incubated in a static incubator for 48 h at 30 °C. (**b**) Spot plate assays comparing the susceptibility of *tlo*Δ*-TAC1-5* derivatives expressing either *pTET-TLO*β*2* or *pENO-TLO*β*2*. (**c**) Disc diffusion assays on RPMI-1640 plates containing fluconazole (25 µg ml^−1^) with and without cyclosporine (1 µg ml^−1^) incubated at 30 °C for 48 h.

We next investigated the effect of reintroducing the *TLO* genes on fluconazole susceptibility in the *tlo*Δ and *tlo*Δ-*TAC1-5* backgrounds. Reintroduction of *TLO*α*1*, *TLO*β*2* or *TLO*γ*11* to the *tlo*Δ mutant without the *TAC1-5* allele did not impact significantly the fluconazole MIC (all 1 µg ml^−1^) and did not enable growth on 15 µg ml^−1^ fluconazole spot plates (Fig. 4a). In the *tlo*Δ-*TAC1-5* background, introduction of *pTET-TLO*α*1* restored strong growth in the presence of 15 µg ml^−1^ fluconazole, whereas the weakly expressed *pTET-TLO*β*2* and *pTET-TLO*γ*11* genes exhibited limited growth (Fig. 4a). As previously noted, expression of *TLO*β*2* from the strong *pENO* promoter resulted in phenotypic complementation of the morphological defects of the *tlo*Δ*-TAC1-5* mutant. Analysis of fluconazole susceptibility showed that the *pENO-TLO*β*2* gene could restore growth on 15 µg ml^−1^ fluconazole in the *tlo*Δ*-TAC1-5* mutant to a similar level as *pTET-TLO*α*1* (Fig. 4b).

Disc diffusion assays confirmed the results of the fluconazole spot plate experiments. In the presence of cyclosporine, the *tlo*Δ*-TAC1-5* mutant expressing *TLO*α*1* exhibited the highest level of fluconazole resistance with the smallest inhibition zone, while the weakly expressed *pTET-TLO*β*2* and -*TLO*γ*11* genes exhibited intermediate inhibition zone sizes (Fig. 4c and Table S4). In the absence of cyclosporine, fluconazole tolerance was still apparent in each of the transformants (Fig. 4c). Fluconazole Etest assays were also carried out to measure fluconazole MIC values for *tlo*Δ*-TAC1-5* mutants expressing individual *TLO*s (Table 1). The *tlo*Δ*-TAC1-5* mutant expressing *TLO*α*1* exhibited an MIC value identical to WT-*TAC1-5* (16 µg ml^−1^). The *tlo*Δ*-TAC1-5* mutants expressing *pTET-TLO*β*2* and *pTET-TLO*γ*11* had MIC values of 6–8 µg ml^−1^ which were similar to the parental *tlo*Δ*-TAC1-5* mutant. However, expression of *TLO*β*2* from the strong *pENO1* promoter in the *tlo*Δ*-TAC1-5* background resulted in a similar MIC to the *pTET-TLO*α*1* strain of 16 µg ml^−1^.

Terbinafine susceptibility was also examined using spot plate assays (Fig. 4). Reintroduction of *TLO*α*1* in *tlo*Δ*-TAC1-5* significantly enhanced growth of the strain on 5 µg ml^−1^ terbinafine plates. Restoration of *TLO*β*2* under the control of the *pENO* promoter also restored growth on 5 µg ml^−1^ terbinafine plates to a similar level as *TLO*α*1*, whereas *TLO*γ*11* had no impact on terbinafine susceptibility.

We next tested whether additional representatives of the *TLO* alpha and gamma clades conferred the same phenotypes as *TLO*α*1* and *TLO*γ*11* in the *tlo*Δ*-TAC1-5* background. Similar to *pTET-TLO*α*1*, the alpha clade genes *pTET-TLO*α*3* and *pTET-TLO*α*34* restored WT cellular morphology and hyphal growth in 10% serum in the *tlo*Δ*-TAC1-5* background (Fig S1). *TLO*α*34* was similar to *TLO*α*1* in its ability to restore fluconazole resistance in the *tlo*Δ*-TAC1-5* background (Etest MIC 16 µg ml^−1^ and Fig. S2a). However, *pTET-TLO*α*3*-expressing derivatives exhibited weaker growth on fluconazole spot plates (Fig. S2b) and exhibited a slightly lower fluconazole MIC of 12 µg ml^−1^ in the *tlo*Δ*-TAC1-5* background. We also tested two additional *TLO* gamma clade members, *pTET-TLO*γ*5* and *pTET-TLO*γ*7*. Neither gene restored cellular morphology or hyphal growth (Fig. S1) and had no effect on fluconazole resistance (Etest MIC 6 µg ml^−1^ and Fig. S2). *TLO*α*34* was also able to restore comparable levels of growth as *TLO*α*1* on plates containing 5 µg ml^−1^ terbinafine, whereas *TLO*α*3* expression resulted in an intermediate phenotype (Fig. S3). The strongly expressed *pENO-TLO*β*2* also restored growth on 5 µg ml^−1^ terbinafine, whereas the gamma genes *pTET-TLO*γ*5* and *pTET-TLO*γ*7* did not alter terbinafine susceptibility in the *tlo*Δ*-TAC1-5* background (Fig. S3).

### Global gene expression in *TLO*-complemented *tlo*Δ*-TAC1-5* strains

**Fig. 5.**
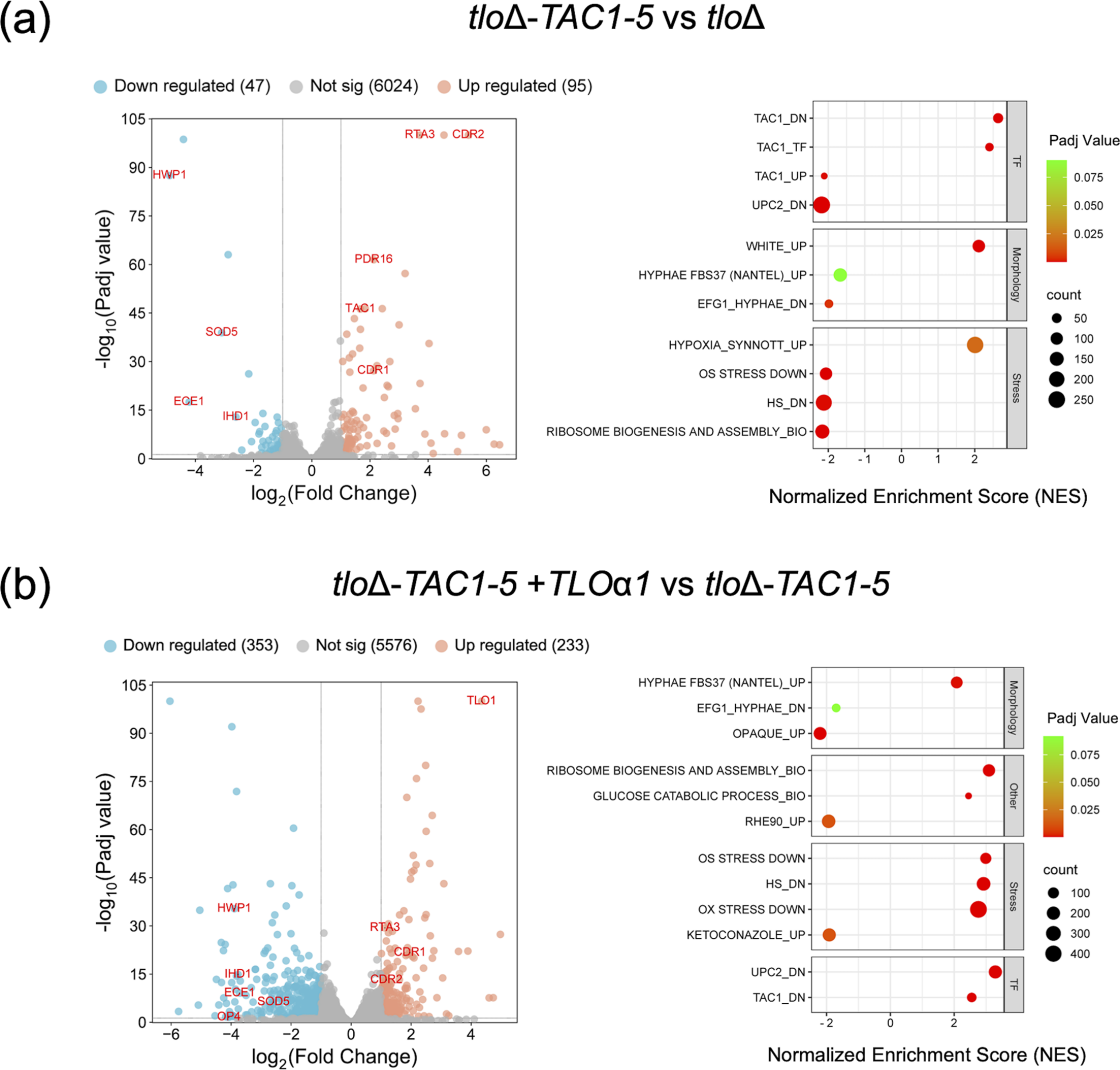
RNAseq analysis of the *tlo*Δ and *tlo*Δ*-TAC1-5* mutants and *tlo*Δ*-TAC1-5* mutant complemented with *TLO*α*1*. (**a**) The left panel shows a volcano plot of significant (Padj <0.05, log_2_FC>1.0) changes in gene expression in *tlo*Δ*-TAC1-5* compared to *tlo*Δ. The right panel shows a plot of representative, non-redundant categories of differentially expressed genes identified by GSEA. Positive NES=increased expression; negative NES=decreased expression. Gene set descriptions can be found in the Supplementary data file. (**b**) Comparison of *tlo*Δ*-TAC1-5+TLO*α*1* versus *tlo*Δ*-TAC1-5* as described in (**a**).

In order to understand the impact of the *TAC1-5* allele on gene expression and to investigate the functionality of the *TLO* genes in this background, we carried out RNAseq analysis on mid-exponential YEPD cultures (Fig. 5 and Supplementary data file). We first compared the transcriptomes of the *tlo*Δ mutant and the *tlo*Δ-*TAC1-5* derivative to investigate the impact of the *TAC1-5* allele on the *tlo*Δ transcriptome. This analysis showed increased expression of *CDR1* and *CDR2* in the *TAC1-5* derivative and also identified increased expression of other Tac1 target genes such as *RTA3* and *PDR16*, as well as the *TAC1* gene itself. Unexpectedly, we observed decreased expression of many hypha-specific genes that are normally overexpressed in the *tlo*Δ mutant background, most significantly those regulated by *EFG1* including *ECE1*, *HWP1*, *IHD1* and *SOD5* (Fig. 5a). Introduction of *TLO*α*1* further enhanced expression of the Tac1-activated genes including *CDR1*, *CDR2* and *RTA3* (Fig. 5b). Comparison of the *TAC1-5*-activated gene set with the *TLO*α*1*-induced gene set showed that 28% of *TAC1-5*-activated genes (157 of 563 genes) exhibited further increases in expression in the presence of *TLO*α*1* (Fig. S4). As expected, based on our previous studies, *TLO*α*1* also further reduced the expression of *EFG1*-regulated genes and opaque-phase gene expression and restored glycolytic gene expression to WT levels [21].

In contrast, the introduction of the weakly expressed *pTET-TLO*β*2* gene in the *tlo*Δ**-***TAC1-5* background resulted in very few changes in gene expression, most likely due to the lower level of expression of Tloβ2 protein (Fig. S5). We observed increased expression of *TLO*β*2* itself (log_2_ FC 1.56) and a significant reduction in *ADH1* expression, corresponding to the integration locus of the *pTET-TLO*β*2* construct.

**Fig. 6.**
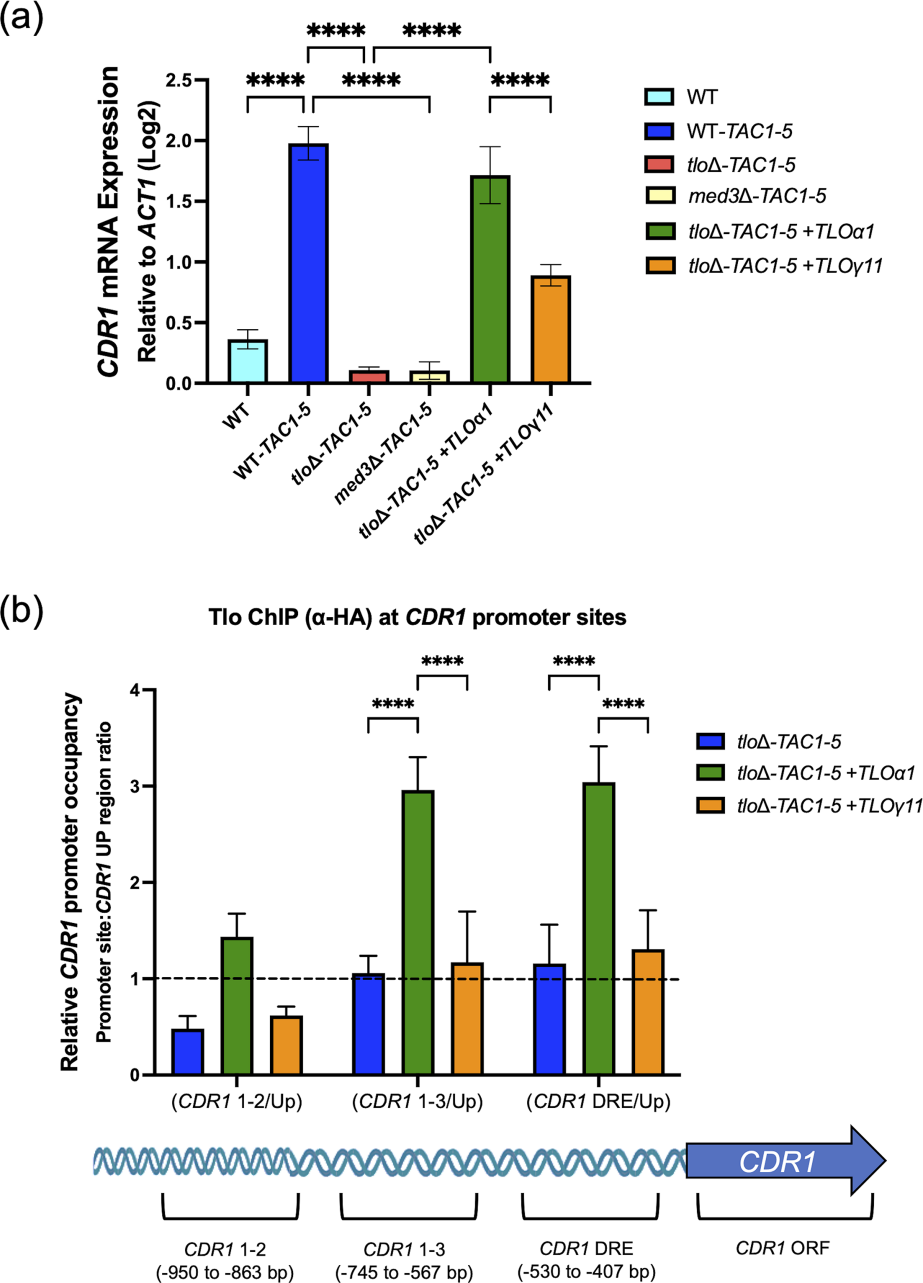
*CDR1* mRNA expression and Tlo protein occupancy across the *CDR1* promoter region in *tlo*Δ*-TAC1-5* mutants complemented with *TLO*α*1* and *TLO*γ*11*. (**a**) *CDR1* expression measured via qRT-PCR in *TAC1-5* derivatives of WT (MAY1244), *tlo*Δ and *med3*Δ and *tlo*Δ*-TAC1-5* mutants complemented with *TLO*α*1* or *TLO*γ*11*. The expression of *CDR1* was normalized relative to *ACT1* and is expressed as log_2_ fold difference. Data represent three biological replicates analysed using one-way ANOVA (***** indicates *P* value<0.0001). (**b**) Tlo occupancy at *CDR1* promoter sites (*CDR1* 1–2, *CDR1* 1–3 and *CDR1* DRE) in *tlo*Δ*-TAC1-5* complemented with *TLO*α*1* or *TLO*γ*11* determined by qPCR following anti-HA antibody ChIP of HA-tagged Tlo proteins. HA-tagged Tlo protein signals (IP/Input) at the three sites were normalized to an internal negative control region (IP/IN at *CDR1* Up) to show relative occupancy; a ratio of 1 was therefore set as the cut-off value to determine positive occupancy (positive=>1). Data represent three biological replicates analysed using one-way ANOVA (*** indicates *P* value<0.001, and **** indicates *P* value<0.0001).

Levels of *CDR1* mRNA in the *tlo*Δ-*TAC1-5* mutant and *TLO*-expressing derivatives were confirmed using qRT-PCR (Fig. 6a). The reintroduction of *TLO*α*1* in the *tlo*Δ**-***TAC1-5* mutant restored *TAC1-5*-activated *CDR1* expression to a similar level as that observed in WT-*TAC1-5*. The reintroduction of the weakly expressed *pTET-TLO*γ*11* only partially restored the expression of *CDR1* activation by *TAC1-5* to levels that were significantly lower than those observed in the *TLO*α*1*-complemented *tlo*Δ**-***TAC1-5* and WT-*TAC1-5* (Fig. 6a).

### Tloα1 localizes to the DRE in the *CDR1* promoter

ChIP-qPCR was performed for HA-tagged Tlo to detect occupancy across the *CDR1* promoter region (Fig. 6b). Three upstream *CDR1* promoter sites were tested; *CDR1* 1–2, *CDR1* 1–3 and *CDR1* DRE (Fig. 6b). An upstream *CDR1* promoter site (*CDR1* Up), previously shown to lack any Mediator binding sites [13], was used as an internal negative control. Extracts from WT-*TAC1-5* were chosen as a negative ChIP control since they did not express any HA-tag. Occupancy at each site was expressed as a ratio to *CDR1* Up levels and ratios higher than 1 were considered positive enrichment. ChIP-qPCR analysis revealed that Tloα1 from *tlo*Δ*-TAC1-5**+**TLO*α*1* showed significant occupancy at the promoter site *CDR1* 1–3 and *CDR1* DRE as it showed significantly higher ratios than WT-*TAC1-*5 (~1). Tloγ11, which we could not detect by Western blot analysis, did not exhibit any significant occupancy at the *CDR1* 1–3 and *CDR1* DRE sites compared to WT-*TAC1-*5. None of the Tlo proteins exhibited significant occupancy at promoter site *CDR1* 1–2, with Tloα1 showing a slightly increased ratio relative to *CDR1* Up.

## Discussion

We have previously shown that the massively expanded *TLO* gene family, which encodes the Med2 component of the Mediator tail module [27], is involved in a wide range of cellular processes in *C. albicans* including responses to antifungal drugs [19,21]. The current study aimed to specifically investigate the role of the *TLO* gene family members in Tac1-activated expression of the *CDR1* gene, which encodes an azole antifungal efflux pump and represents one of the most common mechanisms of azole resistance in *C. albicans*. Previous studies have shown that *CDR1* mRNA expression is highly dependent on the Mediator tail components Med3 and Med15 [13]. The role of the Tlo component of the Mediator tail in this Tac1-mediated regulation of *CDR1* expression is of particular interest due to the expansion of the *TLO* gene family (i.e. there are 14 *TLO* genes, divided into three distinct clades, in *C. albicans* SC5314). We and others have previously shown that there are functional differences between the three *TLO* gene family clades [18,19], suggesting that individual Tlo proteins may interact differently with Tac1 or Mediator under specific conditions or perhaps with different affinities.

As expected from our previous studies, the *tlo*Δ, a null mutant in which all 14 copies of *TLO* have been deleted, phenocopies the *med3*Δ Mediator tail mutant [13]. Mutation of *TAC1* using CRISPR-Cas9 mutagenesis to generate a homozygous hyperactive *TAC1-5* allele in the *tlo*Δ and *med3*Δ mutant backgrounds resulted in reduced fluconazole susceptibility compared to WT strains expressing *TAC1-5* (Fig. 1). Reduced levels of *CDR1* mRNA expression in *C. albicans* WT MAY1244 and the two Mediator tail null mutants, *tlo*Δ and *med3*Δ, confirm that basal *CDR1* mRNA expression requires a functional Mediator tail. Deletion of *tlo*Δ and *med3*Δ also prevented Tac1-mediated *CDR1* induction by fluphenazine, indicating that *TLO* genes likely play a major role in the control of *CDR1* expression. Neither of the Mediator tail null mutants we tested significantly affected the basal *CDR2* mRNA expression, and fluphenazine was unable to activate strong *CDR2* mRNA expression (Fig. 2), supporting recent studies showing that *CDR2* expression is highly dependent on chromatin remodelling activity (Swi/Snf) rather than the Mediator tail module [28]. These findings also support the hypothesis that Tac1^GOF^ mutants and xenobiotic-induced WT Tac1 activate *CDR1* transcription through a similar Mediator-dependent mechanism, supporting a potential antifungal strategy to block Tac1 hyperactivation via small molecule inhibitors [13,29].

Introduction of the hyperactive *TAC1-5* allele in the *tlo*Δ background provided some additional insights as our data indicate that even in the absence of *TLO* genes, the hyperactive *TAC1-5* allele still exhibited significant functionality. Introduction of *TAC1-5* into the *tlo*Δ mutant increased *CDR1* and *CDR2* expression, as seen by qRT-PCR and RNAseq analysis, albeit to lower levels than in a WT *TAC1-5* strain. Despite the relatively low levels of *CDR1* and *CDR2* mRNA, the *tlo*Δ*-TAC1-5* strain exhibited a significant increase in fluconazole MIC (~8-fold), which may not be explained solely by *CDR1* and *CDR2* mRNA levels. RNAseq analysis of the *tlo*Δ*-TAC1-5* strain indicates that the *TAC1-5* allele has wide-ranging impacts on gene expression in the *tlo*Δ background, including a negative impact on the expression of genes that require *UPC2* for expression (UPC2_DN category). Unexpectedly, expression of *TAC1-5* in the *tlo*Δ mutant also reduced fluconazole tolerance (Fig. 1b). O’Connor-Moneley *et al.* [21] recently characterized fluconazole tolerance in the *tlo*Δ mutant and linked this phenotype with reduced expression of *ERG* genes in the *tlo*Δ mutant versus WT strains [21]. The impact of the *TAC1-5* allele on fluconazole susceptibility clearly goes beyond regulation of *CDR1* expression and may involve multiple pathways and targets.

In order to investigate if there is functional diversity within the Tlo family with regards to their ability to facilitate hyperactive Tac1 activity, we used the *pTET* promoter to express representative *TLO* genes from the alpha (*TLO*α*1*, *TLO*α*3* and *TLO*α*34*), beta (*TLO*β*2*) and gamma (*TLO*γ*5*, *TLO*γ*7* and *TLO*γ*11*) clades in the *tlo*Δ*-TAC1-5* strain. We included representatives from the *TLO* gamma clade even though previous studies showed that these genes (i.e. *TLO*γ*5* and *TLO*γ*11*) do not appear to be translated, even when expressed from the strong *pENO* promoter, and did not confer any detectable phenotypic changes in the *tlo*Δ mutant [19]. This study supports the apparent lack of functionality of gamma Tlos, as none of the genes examined here could restore WT cellular morphology or had a significant impact on fluconazole susceptibility. It is possible that gamma *TLO* genes have regulatory functions distinct from alpha and beta *TLO* genes (e.g. production of non-coding regulatory RNAs [30]), and studies to generate specific *TLO* gamma mutants are underway to investigate this.

In contrast to the gamma clade, expression of the single member of the beta clade, *TLO*β*2*, in the *tlo*Δ*-TAC1-5* background could restore filamentous growth in serum and increased resistance to fluconazole in the *tlo*Δ mutant. However, in order to detect these phenotypes, it was necessary to express *TLO*β*2* from the strong *pENO1* promoter. The expression of *pTET-TLO*β*2* mRNA in the *tlo*Δ*-TAC1-5* strain was much lower than that previously observed in the parental *tlo*Δ background and could be related to reduced mRNA stability.

All of the alpha *TLO* genes examined in our study (i.e. *TLO*α*1*, *TLO*α*3* and *TLO*α*34*) restored WT cellular morphology, hyphal growth in 10% serum and enhanced resistance to fluconazole in the *tlo*Δ mutant. Expression of these alpha *TLO* genes resulted in an increase in terbinafine and fluconazole resistance, although *TLO*α*3* induced an intermediate fluconazole resistance phenotype (MIC 12 µg ml^−1^ compared to 16 µg ml^−1^). The weaker *TLO*α*3* phenotypes could be expression-related, as was observed with *TLO*β*2.*

We examined Tloα1 activity in detail and showed that it was strongly expressed, restored WT gene expression and increased *CDR1* mRNA levels, as well as increased fluconazole resistance, similar to the levels observed in the WT-*TAC1-5* strain. ChIP analysis of Tloα1 showed that it localizes to the DRE which is the site of Tac1 binding at the *CDR1* promoter. The *CDR1* 1–3 and *CDR1* DRE regions of the *CDR1* promoter regions exhibited the greatest enrichment in Tloα1 levels, whereas *CDR1* 1–2 (the furthest upstream promoter site examined here) had very little enrichment in all Tlo pulldowns. The *CDR1* 1–3 promoter site includes the SRE2 element, a cis element that has been characterized as a steroid-responding region that can be induced by progesterone and *β*-estradiol [31]. The DRE promoter site has been previously proposed as the binding site of Tac1 to initiate the induction of *CDR1* expression [10,13]. It is currently not clear whether the Tloα1 protein binds directly to the *CDR1* DRE, or if the localization is indirect as it is possible that Tloα1 binds indirectly to the DRE via other Mediator components. Future ChIP experiments will be required to clearly show the direct/indirect nature of Tloα1 binding at the *CDR1* DRE and *CDR1* 1–3 promoter sites and could be used to determine which Mediator components can be recruited by Tloα1 at the *CDR1* DRE promoter site.

To summarize, we have shown that individual alpha and beta *TLO* genes can restore fluconazole resistance in a hyperactive Tac1-5 strain where all *TLO* genes have been deleted. This supports the hypothesis that Tlo protein is an important component of the Mediator tail and, for the first time, directly links Tlo as a requirement for the full activity of a transcriptional regulator. This study also suggests functional diversity in the ability of the Tlo family to activate transcription, providing further evidence that the function of the Tlo gamma sub-family has diverged from encoding canonical Mediator tail polypeptide components. Future studies should determine whether there is diversity in Tac1 interactions within the alpha and beta families, which could impact on the development of antifungal resistance.

## Supplementary material

10.1099/mic.0.001594 Uncited Fig. S1.

10.1099/mic.0.001594 Uncited Fig. S2.

## Abbreviations

AAD: acidic activation domain
ABC: ATP-binding cassette
DBD: DNA-binding domain
DRE: drug response element
FA: formaldehyde-assisted
GOF: gain-of-function
GOF: gain-of-function
GSEA: Gene Set Enrichment Analysis
IN: input
MHR: middle homology region
PIs: protease inhibitors
TBP: TATA-binding protein
YEPD: yeast extract peptone dextrose
