# Supplementary material for: Fluconazole resistance and CDR1 expression in Candida albicans mediated by the hyperactive Tac1-5 transcriptional activator requires Tlo proteins
Source: Microbiology (Reading). 2025 Sep 1;171(9):001594. doi: 10.1099/mic.0.001594 (PMC12401524; doi:10.1099/mic.0.001594)
Supplement: Uncited Fig. S1. [file mic-171-01594-s001.pdf]

Table S1. ***C. albicans*** **strains used in this study**

Strain	Genotype*	**Parent**	**Source**
MAY1244	*leu2Δ/LEU2*	SC5314	(Fletcher *et al.*, 2023)
med3Δ	*med3Δ/med3Δ, leu2Δ/LEU2*	MAY1244	(Fletcher *et al.*, 2023)
tloΔ (CC16)	*tloΔ, leu2Δ/LEU2*	AHY940	(Fletcher *et al.*, 2023)
tloΔ +TLOα1	*tloΔ, ADH1/adh1::pTET-TLOα1, leu2Δ/LEU2*	*tloΔ* (CC16)	(Fletcher *et al.*, 2023)
*tloΔ +TLO*β2	*tloΔ, ADH1/adh1::pTET-TLOβ2, leu2Δ/LEU2*	*tloΔ* (CC16)	(Fletcher *et al.*, 2023)
tloΔ +TLOγ11	*tloΔ, ADH1/adh1::pTET-TLOγ11, leu2Δ/LEU2*	*tloΔ* (CC16)	(Fletcher *et al.*, 2023)
WT-*TAC1-5*	*TAC1-5/TAC1-5, leu2Δ/LEU2*	MAY1244	This study
med3Δ-TAC1-5	*med3Δ/med3Δ, TAC1-5/TAC1-5, leu2Δ/LEU2*	*med3Δ*	This study
tloΔ -TAC1-5	*tloΔ, TAC1-5/TAC1-5, leu2Δ/LEU2*	*tloΔ* (CC16)	This study
*tloΔ -TAC1-5 +TLO*α*1*	*tloΔ, ADH1/adh1::pTET-TLOα1,*<br>*TAC1-5/TAC1-5, leu2Δ/LEU2*	*tloΔ-TAC1-5*	This study
*tloΔ -TAC1-5 +TLO*β2	*tloΔ, ADH1/adh1::pTET-TLO*β2,<br>*TAC1-5/TAC1-5, leu2Δ/LEU2*	*tloΔ-TAC1-5*	This study
*tloΔ -TAC1-5 +TLO*γ11	*tloΔ, ADH1/adh1::pTET-TLOγ11*,<br>*TAC1-5/TAC1-5, leu2Δ/LEU2*	*tloΔ-TAC1-5*	This study
*tloΔ-TAC1-5+pENO-TLO*β2	*tloΔ, tlo34Δ/tlo34::pENO-TLO*β2,<br>*TAC1/TAC1-5, leu2Δ/LEU2*	*tloΔ-TAC1-5*	This study
*tloΔ -TAC1-5 +TLO*α3	*tloΔ, ADH1/adh1::pTET-TLO*α3,<br>*TAC1-5/TAC1-5, leu2Δ/LEU2*	*tloΔ-TAC1-5*	This study
*tloΔ -TAC1-5 +TLO*α34	*tloΔ, ADH1/adh1::pTET-TLO*α34,<br>*TAC1-5/TAC1-5, leu2Δ/LEU2*	*tloΔ-TAC1-5*	This study
*tloΔ -TAC1-5 +TLO*γ5	*tloΔ, ADH1/adh1::pTET-TLO*γ5,<br>*TAC1-5/TAC1-5, leu2Δ/LEU2*	*tloΔ-TAC1-5*	This study
*tloΔ -TAC1-5 +TLO*γ7	*tloΔ, ADH1/adh1::pTET-TLO*γ7,<br>*TAC1-5/TAC1-5, leu2Δ/LEU2*	*tloΔ-TAC1-5*	This study

*The Δ*tlo* genotype indicates deletion in all 14 members of the *TLO* gene family

Table S2. Sequences of oligonucleotides used in this study

Name	Sequence (5'-3')
**qRT-PCR**	
ACT1 F	AGCTCCAGAAGCTTTGTTCAGACCAG
ACT1 R	TGCATACGTTCAGCAATACCTGGG
RT CDR1 F	TGTGCTGAACGTGAATATGTTT
RT CDR1 R	TTGGTGGAACTCATTTGACAG
RT CDR2 F	TTGTGCACCTAGAGAATTGGTT
RT CDR2 R	TTGACTAAACAAAGCATTGATG
CaTLO1 qPCR F	ACTAGCCCCAACAACGAACT
CaTLO1 qPCR R	CATAACGCCGAGACACCACT
CaTLO2 qPCR F	TCAACGACATGCAGAACGAC
CaTLO2 qPCR R	TCATGTCCAAGTCGCTGTCT
CaTLO11 qPCR F	ATAACCCAACTGCTCAACGG
CaTLO11 qPCR R	CACTTCTTGGCTTCCTCTGC
***C. albicans* CRISPR**	
AHO1096	GACGGCACGGCCACGCGTTTAAACCGCC
AHO1098	CAAATTAAAAATAGTTTACGCAAG
AHO1097	CCCGCCAGGCGCTGGGGTTTAAACACCG
TAC1-5 gRNA (gRNA underlined)	CGTAAACTATTTTTAATTTGACAATAATTTGGGGATTTAAGTTTTAGAGCTAGAAATAGC
TAC1-5 Repair F	TTTAGTCAATTTAATAATTTACCCAATTTTTTCTTTGACGATAATTTGGGGATTTAAAGT
TAC1-5 Repair R	TATACATCGCTTTCACCAATTACAACTCTTTTTAACACTTTAAATCCCCAAATTATCGT
**TAC1-5 Sanger-sequencing**	
TAC1 ampF	AACTCAACTGGAGAGCCTCAAG
TAC1-5R	AAAGTCATCTTCTTGCTTAC
**pNIM1 insert checks**	
AAK_29	ACCCATTATTGTCGACATGTC
AAK_31	TAGCTTCTGGGCGAGTTTAC
Pan TLO	GACCAGATATTGAAATCATC
Tlo tag (*Bgl*II) R	ATGCAGATCTACCTAAGCGTAATCTGGAAC

Table S3. Zone of inhibition measurements (mm) for the indicated strains when incubated with fluconazole discs (25 µg/ml) for 48 h at 30˚C, showing diameter (mm) and standard deviation (Std Dev) of three replicate experiments.

	MAY1244 (WT)	WT-*TAC1-5*	*tlo*Δ	*tlo*Δ-*TAC1-5*	*med3*Δ	*med3*Δ-*TAC1-5*
Mean	29.3	15.3	30.3	18.5	30.2	18.3
Std Dev	0.6	1.5	0.6	1.3	1.3	1.9

Table S4. Zone of inhibition measurements (mm) for the indicated strains when incubated with fluconazole discs (25 µg/ml) for 48 h at 30˚C, showing mean diameter (mm) and standard deviation (Std Dev) of three replicate experiments.

	*tlo*Δ	*tlo*Δ-*TAC1-5*	*tlo*Δ-*TAC1-5* +*pTET-TLO*α*1*	*tlo*Δ-*TAC1-5* +*pTET-TLO*β*2*	*tlo*Δ-*TAC1-5* +*pTET-TLO*γ*11*
Mean	30.3	18.5	16.2	17.7	18.7
Std Dev	0.6	1.3	1.2	1.2	1.5

Fig. S1

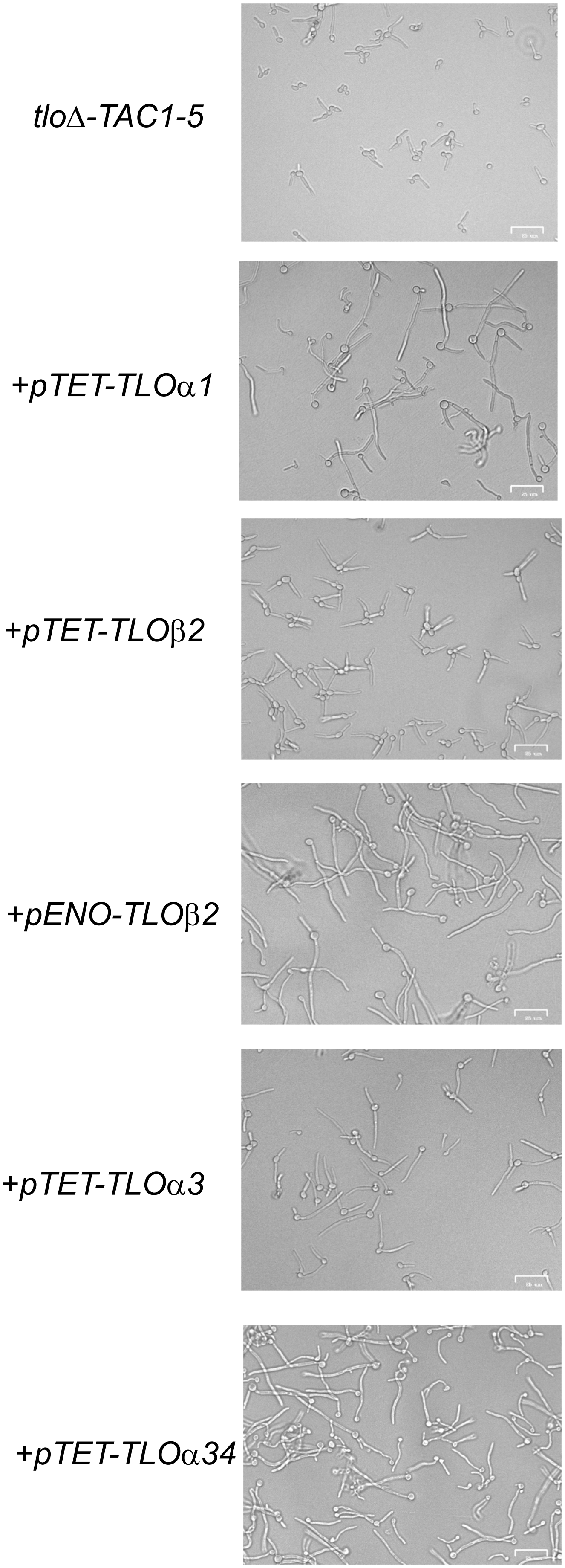

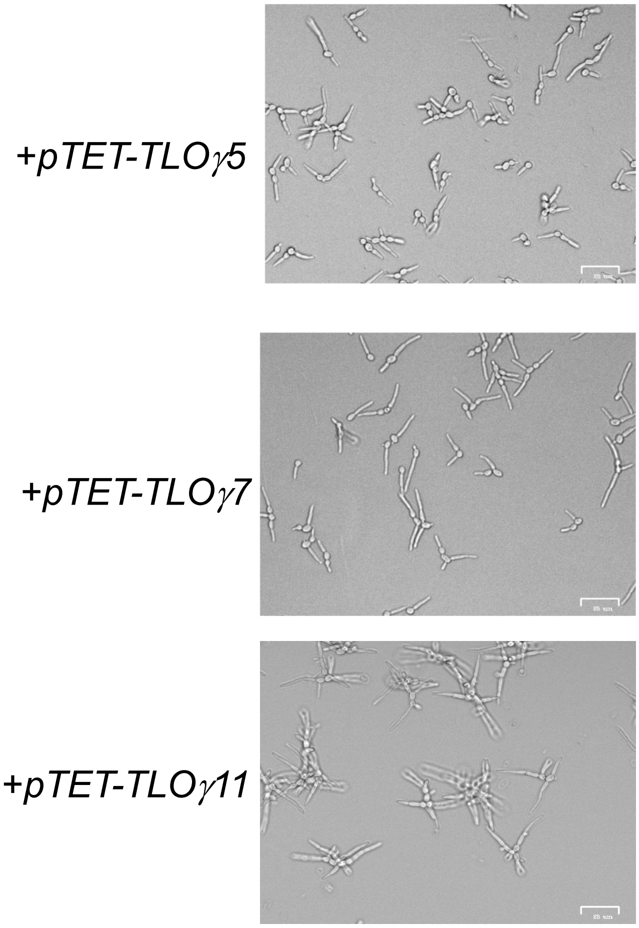

Figure S1. Morphology of *tlo*Δ-*TAC1-5* and derivatives complemented with the indicated *TLO* genes in RPMI-1640 supplemented with 10% serum. Cells from overnight YEPD cultures were used to inoculate RPMI-10% serum ($2x10^6$ cells) and incubated at 37˚C for 3 hours. Cells were visualized at 20x under Zeiss Axio microscope.

Fig. S2

(a)

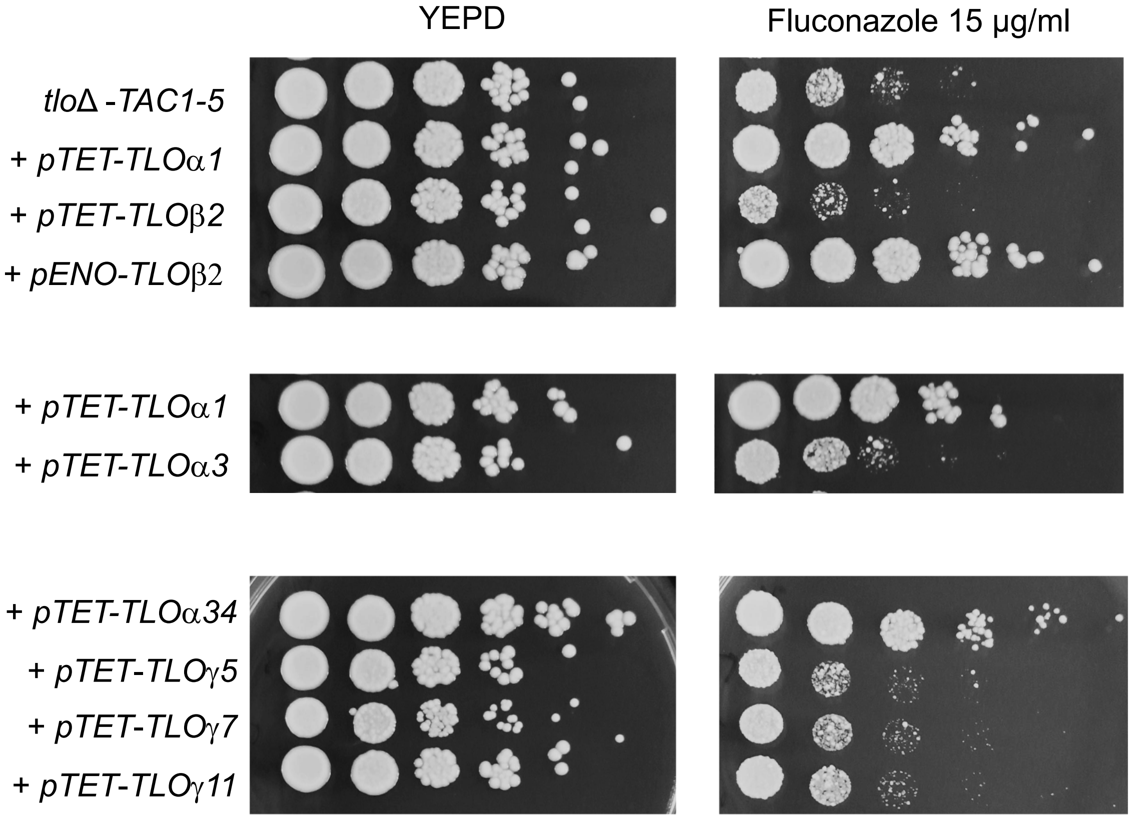

(b)

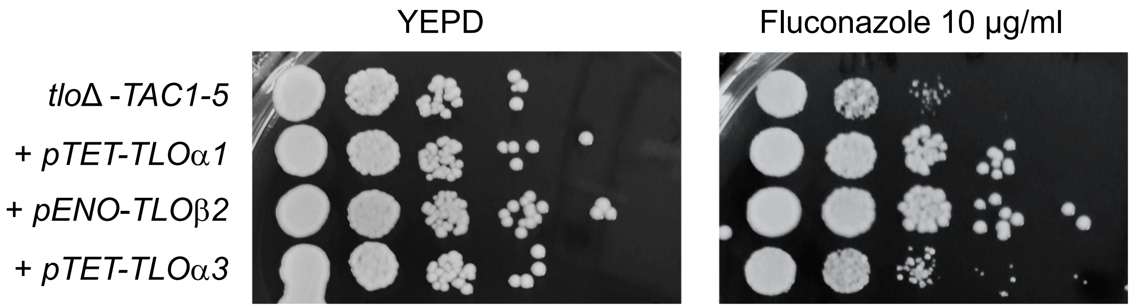

Figure S2. Fluconazole susceptibility of *tlo*Δ-*TAC1-5* and derivatives complemented with the indicated *TLO* genes. Fluconazole spot plate assays were inoculated with serial dilutions of overnight cultures (2x10^6 to 2x10^2 cells/ml) on YEPD plates with or without fluconazole (10 µg/ml or 15 µg/ml). Plates were incubated in a static incubator for 48 h at 30°C.

Fig. S3

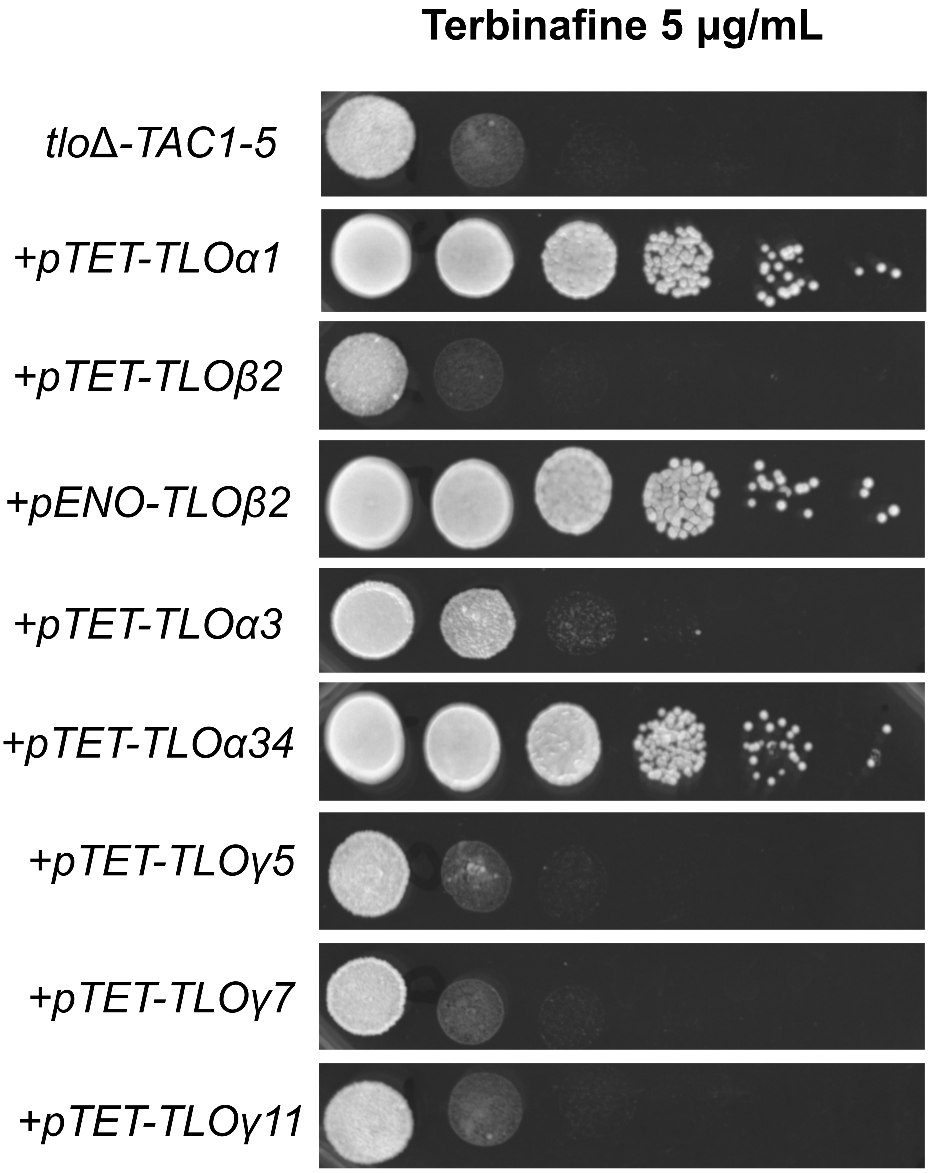

Figure S3. Terbinafine susceptibility of *tlo*Δ-*TAC1-5* and derivatives complemented with the indicated *TLO* genes. Terbinafine spot plate assays were inoculated with serial dilutions of overnight cultures ($2x10^6$ to $2x10^2$ cells/ml) on YEPD plates with or without terbinafine (5 µg/ml). Plates were incubated in a static incubator for 48 h at 30°C.

Fig. S4

Upregulated genes

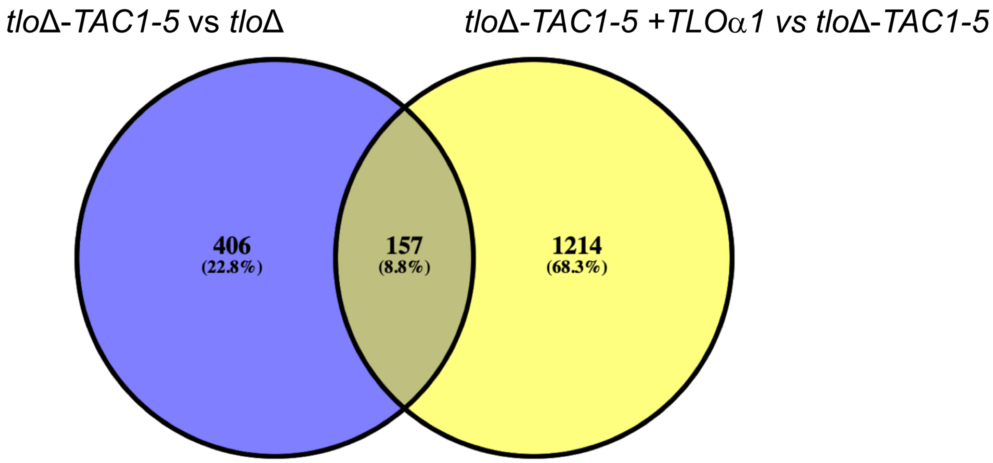

Downregulated genes

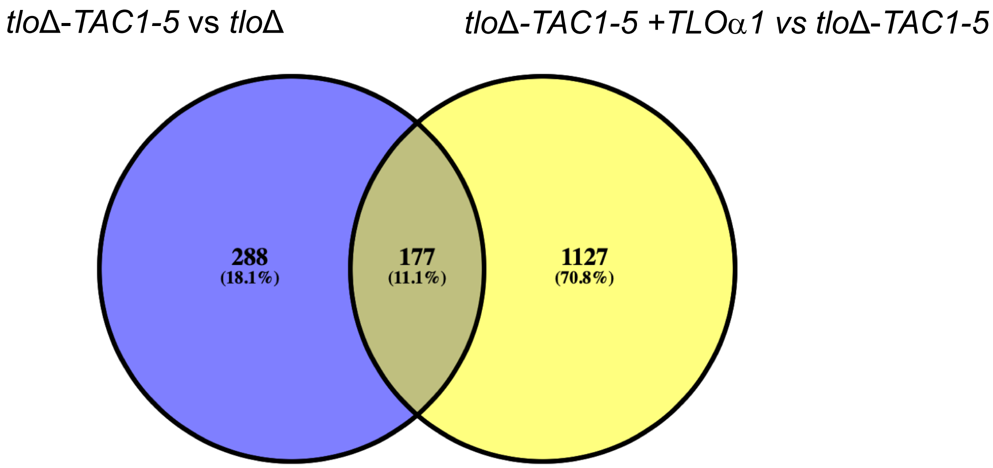

Figure S4. Venn diagrams comparing gene expression in *tlo*Δ-*TAC1-5* and *tlo*Δ-*TAC1-5* +*TLOα1* strains. Upper panel shows common upregulated genes (Padj <0.05) in the comparisons "*tlo*Δ-*TAC1-5* vs *tlo*Δ" and "*tlo*Δ-*TAC1-5 +TLOα1 vs tlo*Δ-*TAC1-5"*. Lower panel shows common downregulated genes (Padj <0.05) the comparisons "*tlo*Δ-*TAC1-5* vs *tlo*Δ" and "*tlo*Δ-*TAC1-5 +TLOα1 vs tlo*Δ-*TAC1-5"*.

Fig. S5

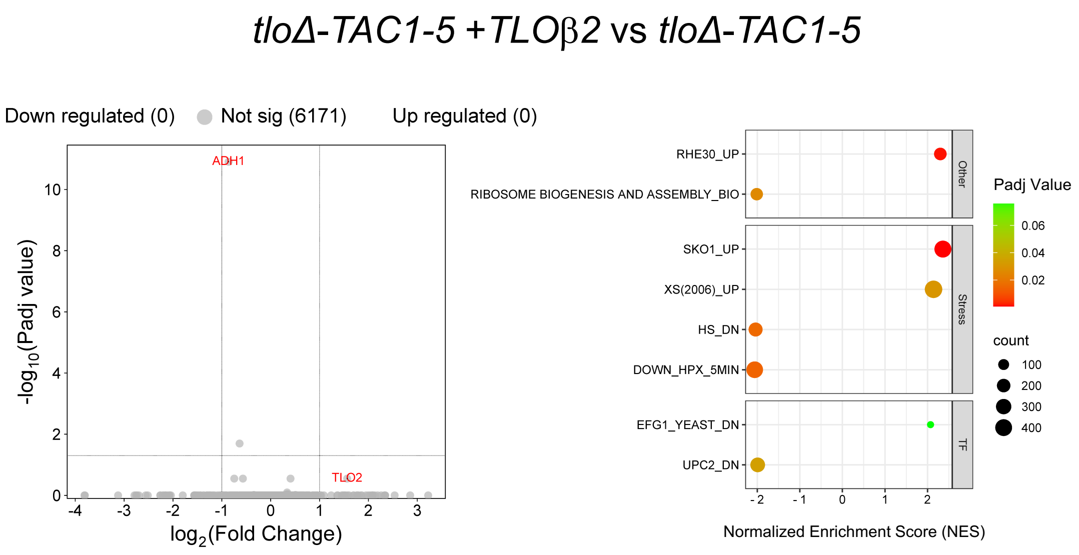

Figure S5. RNA seq analysis of the *tlo*Δ*-TAC1-5* mutant and *tlo*Δ*-TAC1-5* mutants complemented with *TLO*β*2*. Left panel shows volcano plot of significant (Padj <0.05, log2FC >1.0) changes in gene expression in *tlo*Δ-*TAC1-5* compared to *tlo*Δ. Right panel shows plot of representative, non-redundant categories of differentially expressed genes identified by Gene Set Enrichment Analysis (GSEA). Positive NES = increased expression, negative NES = decreased expression.
